# The Role of Short-Chain Fatty Acids in Metabolic Dysfunction-Associated Steatotic Liver Disease and Other Metabolic Diseases

**DOI:** 10.3390/biom15040469

**Published:** 2025-03-22

**Authors:** Eliane Münte, Phillipp Hartmann

**Affiliations:** 1 Department of Pediatrics, University of California San Diego, 9500 Gilman Dr, La Jolla, CA 92093, USA; 2 Division of Gastroenterology, Hepatology & Nutrition, Rady Children’s Hospital San Diego, San Diego, CA 92123, USA

**Keywords:** gut microbiota, MASLD, obesity, SCFAs, T2DM

## Abstract

With its increasing prevalence, metabolic dysfunction-associated steatotic liver disease (MASLD) has emerged as a major global public health concern over the past few decades. Growing evidence has proposed the microbiota-derived metabolites short-chain fatty acids (SCFAs) as a potential factor in the pathophysiology of MASLD and related metabolic conditions, such as obesity and type 2 diabetes mellitus (T2DM). By influencing key pathways involved in energy homeostasis, insulin sensitivity, and inflammation, SCFAs play an important role in gut microbiota composition, intestinal barrier function, immune modulation, and direct metabolic signaling. Furthermore, recent animal and human studies on therapeutic strategies targeting SCFAs demonstrate their potential for treating these metabolic disorders.

## 1. Introduction

Short-chain fatty acids (SCFAs) are a group of saturated aliphatic acids with fewer than six carbon atoms, including acetate (C2), propionate (C3), and butyrate (C4) [1]. They are primarily produced through microbial fermentation of dietary fibers in the gut and play a crucial role in gut health, energy metabolism, and immune regulation [2,3]. SCFAs serve as key signaling molecules influencing host physiology by modulating inflammatory responses, maintaining intestinal barrier integrity, and regulating metabolic pathways [4]. Their impact extends beyond the gut, with emerging research linking SCFAs to systemic effects on metabolism, neurobiology, and chronic disease [5].

Metabolic dysfunction-associated steatotic liver disease (MASLD), previously known as nonalcoholic fatty liver disease (NAFLD), is a spectrum of liver conditions characterized by excessive fat accumulation in the liver, ranging from simple hepatic steatosis to more severe forms, such as metabolic dysfunction-associated steatohepatitis (MASH), formerly termed nonalcoholic steatohepatitis (NASH), which can eventually lead to liver fibrosis, cirrhosis, and hepatocellular carcinoma (HCC) [6,7,8]. Furthermore, the recent change in nomenclature also led to a change in the definition of MASLD to include the presence of at least one of five cardiometabolic risk factors, namely (1) body mass index (BMI) ≥ 25 kg/m^2^ or waist circumference (WC) > 94 cm (male) or > 80 cm (female); (2) fasting serum glucose ≥ 100 mg/dL or HbA1C ≥ 5.7% or diagnosis of type 2 diabetes mellitus (T2DM) or treatment for T2DM; (3) blood pressure ≥ 130/85 mmHg or specific antihypertensive drug treatment; (4) plasma triglycerides ≥ 150 mg/dL or lipid-lowering treatment; and (5) plasma HDL-cholesterol ≤ 40 mg/dL (male) or ≤50 mg/dL (female) or lipid-lowering treatment [7,9,10,11].

Over the past few decades, MASLD has emerged as a major global public health concern, with its prevalence and annual direct medical costs steadily increasing [12,13,14]. A recent meta-analysis by Younossi et al. based on MASLD studies from 1990 to 2019 revealed that the global prevalence of MASLD increased by over 50% from 25.26% in 1990–2006 to 38.00% in 2016–2019 [12]. Similar but lower increases by 26% from 3.73% to 4.71% global prevalence were also reported in pediatric MASLD during the same time frame [13]. Moreover, MASLD has become a major cause of chronic liver disease (CLD), with approximately one-quarter of subjects with simple steatosis progressing to MASH, and again, more than a quarter of those developing severe fibrosis [15,16,17,18]. From 2016 to 2019, CLD moved from being the twelfth leading cause of death to the tenth leading cause of death [19].

In recent years, the human microbiome and the gut–liver axis have also come into focus in scientific research, with studies suggesting the involvement of short-chain fatty acids (SCFAs), a product of bacterial fermentation in the gut, in various cellular processes [20,21].

In this context, this review aims to provide detailed information on MASLD and SCFAs in general, as well as their role in MASLD and other metabolic diseases associated with MASLD.

## 2. Metabolic Dysfunction-Associated Steatotic Liver Disease

### 2.1. Pathogenesis and Progression of Metabolic Dysfunction-Associated Steatotic Liver Disease

The pathogenesis of MASLD is complex and involves an interplay of multiple factors, supporting a “multiple-parallel hit” model [22,23,24,25,26]. Key aspects of MASLD pathogenesis include lipid accumulation and insulin resistance. Insulin resistance reduces glucose uptake by muscle and adipose tissue, leading to peripheral lipolysis, which in turn raises the liver’s exposure to both glucose and free fatty acids. Likewise, excessive caloric intake results in higher levels of free fatty acids in the bloodstream, leading to increased ectopic fat accumulation, including in hepatocytes [23,25]. Free fatty acids are not only stored in the liver in the form of triglycerides but can also directly generate oxidative stress and activate inflammatory pathways in the liver. High levels of free fatty acids overload the mitochondria, where β-oxidation normally takes place, thus leading to the generation of reactive oxygen species (ROS). These ROS drive oxidative stress, trigger inflammatory pathways, and cause mitochondrial damage, which reduces the proliferation rate of hepatocytes, resulting in reduced endogenous liver repair [27]. Further, hyperinsulinemia and hyperlipidemia can trigger endoplasmic reticulum (ER) stress, activating pathways that lead to inflammation and apoptosis [28]. Additionally, damaged hepatocytes release extracellular vesicles, inflammatory cytokines, and damage-associated molecular patterns (DAMPs), activating Kupffer cells and promoting inflammation [25,27]. Another important aspect in the pathogenesis of MASLD is gut microbiota dysbiosis, or a microbial imbalance with reduced concentrations of beneficial microbes and an increased abundance of potentially pathogenic microbes [21,29,30]. Dysbiosis contributes to the development of MASLD by disrupting gut–liver homeostasis. This includes compromising the gut barrier, facilitating the portal transport of microbial products including lipopolysaccharides (LPS) to the liver, altering bile acid (BA) profiles, and reducing short-chain fatty acid (SCFA) levels [31,32]. Aside from environmental and other factors, genetic predisposition plays a role in the pathogenesis of MASLD. A twin study revealed that both hepatic steatosis and hepatic fibrosis in MASLD are heritable traits [33]. For example, variations in genes related to hepatic lipid metabolism, such as patatin-like phospholipase domain-containing protein 3 (PNPLA3), transmembrane 6 superfamily member 2 (TM6SF2), membrane-bound O-acyltransferase domain-containing protein 7 (MBOAT7), and glucokinase regulatory protein (GCKR), are associated with increased susceptibility to MASLD [34].

### 2.2. Metabolic Comorbidities Associated with Metabolic Dysfunction-Associated Steatotic Liver Disease

MASLD is closely associated with an increased risk of cardiovascular disease (CVD), with evidence linking it to both fatal and non-fatal cardiovascular events [35,36,37]. A meta-analysis from 2016 including 16 studies revealed that individuals with MASLD had a 64% higher risk of experiencing cardiovascular events, whether fatal or non-fatal, compared with those without the disease. Further, more severe forms of MASLD were associated with an even greater risk [35]. Additionally, MASLD has been linked to several markers of subclinical atherosclerosis, including increased carotid artery intimal-medial thickness, reduced flow-mediated vasodilation, and increased coronary artery calcification, suggesting a heightened burden of early-stage cardiovascular damage [38]. Additional studies have also found a higher prevalence of clinically manifest CVD, including high-risk coronary plaques (59.3% in patients with MASLD vs. 19.0% in patients without MASLD), in patients with MASLD, independent of traditional CVD risk factors [39,40,41]. As a result, MASLD is now recognized as a significant risk factor for major adverse cardiovascular events (MACE), which are the leading cause of mortality in affected individuals [42,43].

The relationship between type 2 diabetes mellitus (T2DM) and MASLD, including MASH, has recently been described as bidirectional and is increasingly recognized as a critical aspect of their pathophysiology [44,45,46,47]. Hence, T2DM not only accelerates the progression of MASLD, but MASLD also heightens the risk of developing T2DM and worsens glycemic control in individuals already diagnosed with the disease [45,46]. A recent globally representative meta-analysis involving nearly 50,000 patients revealed that the prevalence of MASLD among those with T2DM was over two times higher than in the general population (55.5%), with rates of MASH and advanced fibrosis reaching 37.3% and 17%, respectively [48]. Also, T2DM was shown to be a significant risk factor for the development of severe hepatic complications, such as cirrhosis and liver-related mortality [46,49]. Patients with MASH, on the other hand, had a two times higher risk of developing T2DM, and the severity of hepatic steatosis and liver fibrosis was shown to correlate with an increased risk of developing T2DM [50]. Lastly, a study by Sung et al. evaluating the combined influence of insulin resistance, obesity, and MASLD on the risk of T2DM showed a 14-fold increased risk of T2DM when all three factors were present simultaneously [51].

Another major contributor to the development and progression of MASLD is obesity. The relationship between obesity and MASLD is largely mediated by excess adiposity, which promotes hepatic fat accumulation and insulin resistance. Diet plays a critical role in this process, with high consumption of sugary foods, refined carbohydrates, and caloric excess being key drivers of hepatic steatosis [52,53]. Recent studies have shown that MASLD is present in about 75% of individuals with overweight and obesity [54].

Furthermore, MASLD is associated with an increased risk of HCC and various extrahepatic cancers [37,55]. A comprehensive meta-analysis of 10 cohort studies involving more than 180,000 individuals and approximately 8500 incident cases of extrahepatic cancers demonstrated that MASLD significantly raises the risk of developing several types of cancer, including thyroid cancer, gastrointestinal cancers, and cancers in other systems [56].

### 2.3. Therapeutic Management of Metabolic Dysfunction-Associated Steatotic Liver Disease

Therapeutic strategies for managing MASLD, as well as MASH, focus on addressing both lifestyle factors and pharmacological targets. Weight loss through lifestyle modification is a cornerstone of treatment, with reductions in body weight of more than 10% resulting in a 100% improvement in steatosis, a 90% resolution of MASH, and the reversal of liver fibrosis by at least one stage in 81% of individuals [57,58]. Additionally, weight loss interventions such as bariatric surgery, endoscopic bariatric interventions, and anti-obesity drugs have been shown to improve MASLD [59,60]. Pharmacological treatments for MASLD include insulin sensitizers, such as pioglitazone and metformin; lipid-lowering agents, such as statins and ezetimibe; antioxidants; and medications aimed at modulating liver fat metabolism and inflammation (e.g., aramchol, elafibranor, and obeticholic acid) [61].

Further, glucagon-like peptide-1 receptor agonists (GLP-1RAs), including both short-acting (e.g., exenatide) and long-acting (e.g., liraglutide, semaglutide) formulations, are emerging as effective therapies for MASLD and MASH. These incretin mimetics work by activating GLP-1 receptors, which are widely expressed on various cell types throughout the body, including hepatocytes [62,63,64]. Central GLP-1 receptor activation in regions of the hypothalamus and brainstem promotes satiety and reduces appetite, facilitating weight loss [65,66]. Beyond their central effects, GLP-1RAs directly influence the lipid metabolism by oxidizing fatty acids and reducing their influx into hepatocytes, thereby mitigating hepatic fat accumulation and improving liver lipid metabolism [67,68,69,70]. Furthermore, GLP-1RAs have been shown to reduce lipotoxicity and the lipid stress response in hepatocytes, ultimately preventing or delaying the progression of MASLD [71]. GLP-1RAs have demonstrated efficacy in reversing steatohepatitis and improving liver biochemistry, and there are indications that some might even improve fibrosis [72]. However, semaglutide does not improve MASH-related cirrhosis [73]. Moreover, combination therapies, such as semaglutide with cilofexor and/or firsocostat and semaglutide with cagrilintide, have shown promising results in enhancing improvements in liver steatosis and non-invasive tests of fibrosis. Combination therapies furthermore resulted in greater weight reduction and glycemic control compared with semaglutide monotherapy [74,75]. Furthermore, tirzepatide, a novel GLP-1 and glucose-dependent insulinotropic polypeptide (GIP) receptor agonist used for treating T2DM and promoting weight loss, showed promising effects in patients with MASH and stage 2–3 fibrosis in a recent phase 2 trial [76].

In March 2024, resmetirom (MGL-3196), a liver-directed selective thyroid hormone receptor beta (THR-β) agonist, received accelerated approval in the United States for the treatment of adults with MASH and stage 2 or 3 fibrosis and is to date the only drug approved by the Food and Drug Administration for a subset of MASLD [77]. Since thyroid hormones regulate many processes in the hepatic lipid and cholesterol metabolism, MASLD is thought to arise from a state of hepatic hypothyroidism characterized by diminished thyroid hormone levels in the liver [78]. By selectively targeting THR-β, a receptor highly expressed in the liver and crucial for regulating lipid metabolism and inflammation, resmetirom addresses this pathophysiological abnormality. Resmetirom modulates genes involved in lipid metabolism, enhancing hepatic fat oxidation, reducing lipotoxicity, and thereby decreasing hepatic fat accumulation and inflammation [79,80,81]. In a recent phase 3 trial, patients treated with 80–100 mg resmetirom daily for 52 weeks showed MASH resolution without worsening of fibrosis in 25.9–29.9% of cases, compared with 9.7% in the placebo group, as well as fibrosis improvement by at least one stage without NAS worsening in 24.2–25.9%, as compared with 14.2% in the placebo group [82]. Notably, resmetirom has 28-fold selectivity for THR-β over THR-α, which may help prevent the adverse systemic effects of thyroid hormone excess, such as those on the heart and bones, which are mediated primarily by THR-α [83,84].

## 3. Short-Chain Fatty Acids in Metabolic Disease

### 3.1. Short-Chain Fatty Acids

Short-chain fatty acids (SCFAs) are key metabolites produced by the gut microbiota during the fermentation of dietary fiber and proteins, primarily in the cecum and proximal colon, but can also be produced by bacteria of the skin and vagina [85,86]. Bacteria involved in the production of SCFAs include Clostridial clusters IV and XIVa of Firmicutes, encompassing genera such as *Eubacterium*, *Roseburia*, *Faecalibacterium*, and *Coprococcus* [87,88,89]. SCFAs are organic compounds with a carboxyl group (COOH) bound to a short carbon chain of between one and five atoms and include formate (C1), acetate (C2), propionate (C3), butyrate (C4), and valerate (C5) [1,86,90]. The most abundant SCFAs—acetate (C_2_H_4_O_2_), propionate (C_3_H_6_O_2_), and butyrate (C_4_H_8_O_2_)—account for approximately 95% of SCFAs in the body. Of this 95%, acetate makes up 60%, while propionate and butyrate each account for 20% on average in the healthy adult population [91,92]. They exert a wide range of beneficial effects on human health [85,93]. These include anti-inflammatory, immunoregulatory, anti-obesity, anti-diabetes, and neuroprotective properties, alongside potential cardiovascular and hepatoprotective roles [85]. SCFAs influence gene expression and cellular processes through multiple mechanisms, notably by activating G-protein-coupled receptors (GPCRs) such as the free fatty acid receptors (FFARs) FFAR3 (GPR41) and FFAR2 (GPR43), which are expressed on epithelial cells, immune cells, and adipose tissue [94]. Additionally, SCFAs, particularly butyrate, inhibit histone deacetylases, leading to enhanced histone acetylation and modulation of gene expression [5,95,96,97,98,99,100]. This results in potential biological functions such as inhibiting the growth of pathogens, stimulating water and sodium absorption, decreasing colonic pH, and providing energy to colonic epithelial cells [87,88,89]. These actions contribute to the regulation of immune responses, intestinal barrier function, and metabolism, with implications for conditions such as obesity, insulin resistance, and inflammatory bowel diseases. Furthermore, SCFAs may influence appetite regulation and energy homeostasis [5,20,98,101].

### 3.2. Short-Chain Fatty Acid-Producing Bacteria

SCFAs are primarily produced by anaerobic bacteria in the large intestine through the fermentation of indigestible dietary fibers and resistant starches [102,103]. Throughout the human life cycle, the gut microbiome undergoes substantial changes that impact both the production and diversity of SCFAs. As individuals transition through different life stages, dietary changes—such as the shift from breastfeeding to solid foods—alter the types and amounts of substrates accessible to SCFA-producing bacteria. These dietary modifications play a crucial role in shaping the composition of the microbiota and, consequently, its metabolic outputs [104]. In early life (0–3 years), for instance, the microbiota is characterized by low diversity and primarily dominated by Enterobacteriaceae, which gradually shifts toward Bifidobacteriaceae as the child grows [105,106,107]. Following the transition to solid foods, microbial diversity as well as the abundance of Firmicutes increases, reflecting the dietary shift towards more complex carbohydrates [104,105,108]. In the elderly, however, the microbiota shifts again, with Enterobacteriaceae becoming more prevalent [109]. These dynamic changes in the microbiome are mirrored in SCFA production, with varying concentrations of acetate, propionate, and butyrate [106].

**Figure 1:**
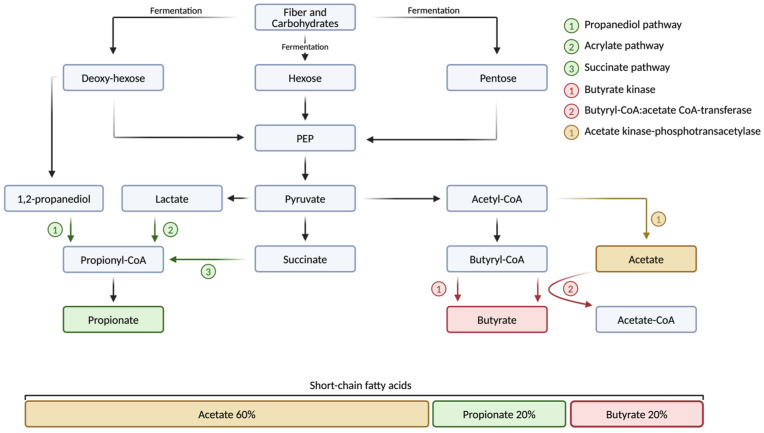
Production of short-chain fatty acids by the human gut microbiota. Dietary fiber and complex carbohydrates, which are indigestible by the human gut, are fermented by the gut microbiota and broken down into monosaccharides, such as hexoses and pentoses. These monosaccharides are further processed through various metabolic pathways. The production of acetate involves the enzymes acetate kinase and phosphotransacetylase, which convert acetyl-CoA into acetyl phosphate, ultimately leading to acetate. The primary pathways for propionate production include the propanediol, acrylate, and succinate pathways. In the production of butyrate, key enzymes such as butyrate kinase and butyryl-CoA:acetate CoA-transferase play crucial roles. Acetate, propionate, and butyrate are the three most abundant short-chain fatty acids (SCFAs) in the human body, collectively accounting for 95% of all SCFAs. Of this 95%, acetate makes up 60%, while propionate and butyrate each account for 20% on average in healthy adults. Acetate-CoA, acetate coenzyme A; acetyl-CoA, acetyl coenzyme A; butyryl-CoA, butyryl coenzyme A; PEP, phosphoenol-pyruvate; propionyl-CoA, propionyl coenzyme A. Created with a license from BioRender.com (accessed on 5 March 2025).

Acetate, the most abundant SCFA, accounts for nearly 60% of all SCFAs in the human body and is produced by a variety of gut bacteria, with significant implications for host health (Figure 1). The primary acetate-producing bacteria span several phyla, including Bacteroidetes (e.g., *Bacteroides* and *Prevotella* species), Actinobacteria (e.g., *Bifidobacterium* species), Verrucomicrobia (e.g., *Akkermansia muciniphila*), and Firmicutes (e.g., *Ruminococcus* species) [110,111,112]. Among these, *Bifidobacterium* species, particularly *B. bifidum*, *B. infantis*, and *B. breve*, are especially prominent in early life, where they dominate the infant gut microbiota and produce acetate through the bifid-shunt pathway [111,113]. This pathway, unique to *Bifidobacteria*, is centered around the key enzyme fructose-6-phosphate phosphoketolase and involves the fermentation of various carbohydrates, including resistant starches, polysaccharides, and simple sugars, including human milk oligosaccharides [114]. Moreover, acetate can be produced by bacteria through several additional pathways, which often coexist within the same microorganism. The two main pathways in the production of acetate include the acetate kinase-phosphotransacetylase (AckA-Pta) pathway and the pyruvate:menaquinone oxidoreductase (PoxB/CidC) pathway [112,115,116] (Figure 1). The capacity to generate acetate through various pathways allows the microbiota to adjust to fluctuating environmental conditions and helps to maintain metabolic flexibility [117]. In addition to its role in energy production, acetate plays an important role in maintaining gut health by protecting against infections such as *Escherichia coli* (*E. coli*) and promoting the growth of other SCFA-producing bacteria and thus contributing to gut microbiota diversity and stability [118,119,120,121].

Similar to acetate, propionate is produced by a variety of gut bacteria, with production levels increasing after the cessation of breastfeeding and the introduction of a more diverse diet [122]. In adults, propionate makes up about 20% of SCFAs in the human gut microbiome (Figure 1). Key propionate-producing bacteria are found across multiple phyla, including Bacteroidetes (e.g., *Bacteroides* and *Prevotella* species), Firmicutes (e.g., the Lachnospiraceae family, *Ruminococcus obeum*, *Coprococcus cactus*, and *Veillonella parvula*), Verrucomicrobia (e.g., *Akkermansia muciniphila*), and Negativicutes (e.g., *Megasphaera elsdenii*) [123,124,125]. Propionate is produced through three primary metabolic pathways: the succinate pathway, employed by Bacteroidetes and some Firmicutes to convert succinate to propionate; the acrylate pathway, used by bacteria such as *Coprococcus cactus* to convert lactate into propionate; and the propanediol pathway, which involves bacteria such as *Ruminococcus obeum* that ferment fucose to produce propionate [123,124,125,126,127,128] (Figure 1). While the production of propionate is influenced by several factors, including the availability of substrates and the structure and diversity of the gut microbiome, the consumption of prebiotic fibers such as beta-glucan has also been shown to stimulate propionate production [129,130].

Butyrate is predominantly produced by anaerobic bacteria within the Firmicutes phylum, including key species such as *Faecalibacterium prausnitzii*, *Eubacterium rectale*, *Eubacterium hallii*, *Roseburia* species, *Clostridium butyricum*, *Coprococcus* species, and *Anaerostipes* species [131,132,133]. In this, *Faecalibacterium prausnitzii*, which belongs to the Ruminococcaceae family, is one of the most abundant butyrate-producing bacteria, comprising about 5% of the gut microbiome [134]. In addition, some members of other phyla such as Actinobacteria, Bacteroidetes, Fusobacteria, and Proteobacteria are also capable of producing butyrate [132]. While butyrate production in the gut occurs through several pathways, the primary route for butyrate production is the acetyl-CoA pathway, which involves two enzymes, butyryl-CoA:acetate CoA-transferase and butyrate kinase [135,136,137] (Figure 1). In this context, lactate and acetate, both byproducts of fermenting dietary fibers and resistant starches, serve as substrates for microbial communities, where they are further processed and ultimately converted into butyrate [138] (Figure 1). Butyrate plays a key role in maintaining an anaerobic gut environment, which helps to prevent the establishment of harmful pathogens including *Salmonella* and *E. coli* [131,132,139]. Furthermore, butyrate provides energy for colonocytes and supports the intestinal barrier by increasing tight junction proteins and mucin 2, a glycoprotein in the intestinal mucus layer [140]. Additionally, butyrate’s beneficial effects on metabolic health have been demonstrated in studies where capsaicin supplementation increased the abundance of butyrate-producing bacteria in mice, leading to lower metabolic endotoxemia and reduced body weight gain [141].

Interactions between SCFA-producing bacteria in the gut play a crucial role in shaping microbial communities and enhancing metabolic functions. One of the most significant interactions is cross-feeding, where the production of one SCFA supports the growth of bacteria that produce other SCFAs. For example, acetate produced by *Bifidobacterium* supports the growth of propionate and butyrate producers, while butyrate, in turn, promotes the growth of *Bifidobacterium* species [119]. Lactate produced by *Bifidobacterium*, on the other hand, can be used by bacteria such as *Eubacterium hallii* and *Anaerostipes caccae* to produce butyrate and propionate [125]. Additionally, certain bacterial species engage in cooperative metabolism, such as *Roseburia* and acetogenic species, which collaborate to produce butyrate in the absence of hydrogen [142]. Furthermore, some fibers act as prebiotics, selectively stimulating beneficial bacteria. The fermentation of fibers and resistant starches also lowers gut pH, which affects microbial composition and favors the growth of butyrate-producing bacteria [102]. A recent animal study further demonstrated that prolonged feeding of a high-fat diet resulted in a reduction of bile acid levels, disrupting gut microbiota balance and ultimately contributing to metabolic disorders and obesity [143].

### 3.3. The Role of Short-Chain Fatty Acids in Metabolic Dysfunction-Associated Steatotic Liver Disease

**Figure 2:**
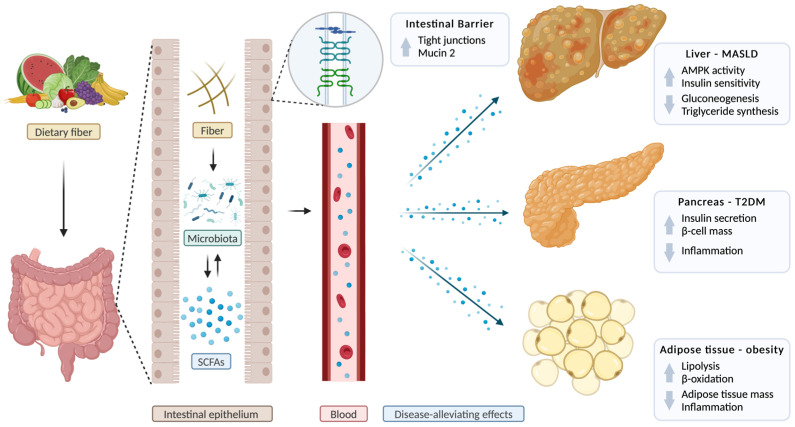
Short-chain fatty acids in metabolic dysfunction-associated steatotic liver disease, type 2 diabetes mellitus, and obesity. Short-chain fatty acids (SCFAs), produced by the gut microbiota from dietary fiber, are transported through the bloodstream to various organs or directly act on the intestinal epithelium. They improve intestinal barrier function by increasing the expression of tight-junction proteins such as claudin-1, claudin-7, zonula occludens-1, zonula occludens-2, and occludin, and by strengthening the intestinal mucus layer by increasing mucin 2 secretion. In the liver, SCFAs improve metabolic dysfunction-associated liver disease (MASLD) by activating 5′ adenosine monophosphate-activated protein kinase (AMPK), a key enzyme that regulates liver metabolism, enhancing insulin sensitivity, reducing gluconeogenesis, and inhibiting fat production. In the pancreas, SCFAs boost insulin secretion, promote β-cell expansion, and reduce inflammation, all of which contribute to the prevention of type 2 diabetes mellitus (T2DM). In adipose tissue, SCFAs reduce inflammation and decrease fat mass by upregulating lipolysis and β-oxidation, thereby helping to alleviate obesity. AMPK, 5′ adenosine monophosphate-activated protein kinase; SCFA, short-chain fatty acids. Created with a license from BioRender.com (accessed on 5 March 2025).

SCFAs play a crucial role in the pathophysiology and potential treatment of MASLD. A recent clinical study involving over 100 individuals, both with and without MASLD, demonstrated significant alterations in gut microbiota composition in the MASLD cohort, with a marked decrease in bacterial richness compared with healthy controls. Namely, four bacterial genera—*Faecalibacterium*, *Subdoligranulum*, *Haemophilus*, and *Roseburia*—were notably reduced across all three MASLD subtypes (mild, moderate, and severe), suggesting a consistent microbial dysbiosis in MASLD. Additionally, SCFAs, including acetic acid and butyric acid, were significantly lower in MASLD patients across all stages, with more pronounced reductions observed in moderate-to-severe cases [144]. In line with these findings, other studies showed a decrease in SCFAs in subjects with MASLD compared with healthy controls [145,146]. Further, a recent clinical trial involving patients with liver steatosis and metabolic disease showed that supplementation of a butyrate-based formula significantly improved liver parameters, such as cholesterol, triglycerides, or gamma-glutamyl transferase, compared with baseline parameters [147] (Table 1). SCFAs influence MASLD pathophysiology through various mechanisms. SCFAs regulate hepatic metabolism by suppressing hepatic gluconeogenesis and lipogenesis, enhancing hepatic uptake of cholesterol, and increasing leptin secretion [148,149,150,151,152,153] (Figure 2). SCFAs also play a role in the regulation of the immune system. Through binding to FFARs, SCFAs regulate the production and release of anti- and pro-inflammatory cytokines and chemokines [154,155,156]. Further mechanisms influencing the inflammatory response include the inhibition of histone deacetylases (HDACs) and the activation of nuclear factor kappa β (NF-κB) in macrophages by butyrate, the production of transforming growth factor beta 1 (TGFβ1), and the reduction of the luminal pH [148,157,158,159,160]. Moreover, SCFAs, in particular butyrate, maintain the homeostasis of the gut barrier and the integrity of gut mucosa by increasing tight junctions, such as claudin-1, claudin-7, zonula occludens-1 (ZO-1), ZO-2, and occludin [161,162,163] (Figure 2). Mucin 2 is the most abundant glycoprotein in the intestinal mucus layer and plays a role in liver disease [164,165]. Butyrate increases the production of mucin 2, strengthening the intestinal mucus layer and preventing toxic substances and inflammatory mediators from entering the bloodstream and finally the liver [148,166,167,168,169] (Figure 2). In recent animal studies investigating the MASH phenotype, administration of SCFAs resulted in a reduction of liver steatosis in diet-induced steatohepatitis [166,170,171]. In humans, vinegar, which is rich in acetate, has been shown to reduce BMI, visceral fat, and serum triglyceride levels [172].

### 3.4. The Role of Short-Chain Fatty Acids in Obesity

SCFAs play a complex and dual role in the context of obesity, with both protective and potentially adverse effects reported in various studies [179]. On the one hand, SCFAs help prevent obesity through multiple mechanisms, including appetite regulation, modulation of lipid and glucose metabolism, and the enhancement of fat oxidation [180,181] (Figure 2). Thus, dietary SCFA supplementation in diet-induced obese mouse models has been shown to increase triglyceride hydrolysis and promote fatty acid oxidation in adipose tissue, contributing to weight management (Figure 2). Additionally, SCFAs stimulate beige adipogenesis, mitochondrial biogenesis, and reduce chronic inflammation, all of which are key processes in obesity prevention [181,182,183,184] (Figure 2). However, excess SCFA production may also contribute to obesity by increasing energy absorption, potentially promoting weight gain [185,186,187]. Furthermore, a human study involving 60 patients with obesity demonstrated that supplementation with 10 g/day of inulin-propionate significantly stimulated postprandial secretion of the satiety hormones peptide YY (PYY) and glucagon-like peptide 1 (GLP-1) compared with inulin controls. This supplementation also non-significantly reduced weight gain and increased weight loss. Additionally, treatment with inulin-propionate significantly decreased intrahepatocellular lipid content, measured using magnetic resonance spectroscopy (MRS), from 22.1% pre-intervention to 15.9% post-intervention in patients with obesity and MASLD [173] (Table 1). Moreover, supplementation with sodium butyrate reduced fat mass (*p* = 0.027), in particular visceral fat (*p* = 0.026), and increased fat-free mass (*p* = 0.032) in adults with obesity [174] (Table 1). In another clinical trial, oral supplementation of butyrate in both healthy individuals and patients with metabolic syndrome was shown to affect immune response. Specifically, it reduced the training capacity of monocytes induced by β-glucan and oxidized low-density lipoprotein [175] (Table 1). Similarly, studies in pediatric patients with obesity showed a significant decrease in BMI by 2.26, BMI *z*-score by 0.31, waist circumference (−5.07 cm), and insulin level (−5.41 μU/mL) when treated with sodium butyrate for 6 months, compared with the placebo-treated group [176] (Table 1). SCFAs may stimulate the release of gut-derived satiety hormones, such as PYY and GLP-1, by binding to the FFAR2 and FFAR3 receptors or directly inhibiting HDAC [188,189]. These hormones influence appetite regulation through several mechanisms: they activate proopiomelanocortin (POMC) neurons in the hypothalamus, suppress neuropeptide Y (NPY), and delay gastric emptying [190,191]. However, the relationship between SCFAs and obesity is not straightforward. Some studies report higher fecal SCFA concentrations in obese individuals, while others show lower levels, indicating a more complex interaction [179,192,193]. De la Cuesta-Zuluaga et al. found a correlation between increased fecal SCFA levels and obesity, metabolic dysregulation, and other comorbidities, while Müller et al. reported that circulating, rather than fecal, SCFAs are associated with metabolic health [194]. Furthermore, obesity is often associated with reduced microbial diversity and dysbiosis, as well as a decline in SCFAs and SCFA-producing bacteria [192]. In obese humans, a diverse microbiota and a high *Akkermansia muciniphila* abundance were linked to the healthiest metabolic status [195]. In an animal model, Ley et al. furthermore demonstrated a reduction in the abundance of Bacteroidetes and an increase in Firmicutes in obese *ob*/*ob* mice compared with lean control mice, highlighting an imbalance in the gut microbiota [196,197]. In another animal study, Cani et al. demonstrated that mice on high-fat diet (HFD) exhibited a decrease in *Bifidobacterium* spp., leading to increased endotoxemia and elevated proinflammatory cytokines [198]. However, excess SCFA production may also contribute to obesity by increasing energy absorption, potentially promoting weight gain [185,186,187]. However, supplementation with SCFAs led to significant alterations in gut microbiota composition, including a reduced Firmicutes/Bacteroidetes ratio [181], which can be viewed as a positive effect, as the Firmicutes/Bacteroidetes ratio is frequently high in obese mice, as stated above [196]. SCFA treatment can also reduce opportunistic pathogen concentrations in vitro and in vivo, such as *Clostridioides difficile*, *E. coli*, *Salmonella enterica*, or *Klebsiella pneumoniae*, among others [199,200]. On the other hand, treatment with SCFAs can also decrease the relative abundance of SCFA-producing microbes (e.g., *Odoribacter*, *Rikenella*) in mice despite the beneficial effects of the SCFA treatment itself [181]. SCFAs influence obesity-related processes through various pathways, including inducing a switch from lipid synthesis to utilization by decreasing peroxisome proliferator-activated receptor gamma (PPARγ) expression and activity, activation of 5′ adenosine monophosphate-activated protein kinase (AMPK) to stimulate oxidative metabolism, and the reduction of cholesterol levels [152,192,201] (Figure 2). Moreover, Sanna et al. demonstrated in a clinical study involving nearly 1000 individuals that increased gut production of butyrate is correlated with improved insulin sensitivity [202]. As mentioned previously, SCFAs exert their functions by activating FFARs. Consistent with this, FFAR2-deficient mice on a normal diet gained weight, whereas FFAR2-overexpressing mice remained lean despite being fed a high-fat diet [203]. However, butyrate and propionate administration in FFAR3-deficient mice still resulted in decreased food intake and body weight [204].

### 3.5. The Role of Short-Chain Fatty Acids in Type 2 Diabetes Mellitus

Type 2 diabetes mellitus (T2DM) is associated with alterations in gut microbiota composition, specifically a decrease in butyrate-producing bacteria, leading to reduced SCFA production. This dysbiosis may contribute to the development and progression of T2DM by impairing key metabolic processes [205,206,207,208,209]. SCFAs, particularly butyrate, play a critical role in improving glucose homeostasis by enhancing insulin sensitivity, reducing fasting insulin concentrations, promoting glucose uptake, and reducing hepatic glucose production [210,211,212] (Figure 2). In a mouse model assessing the impact of acetic acid on glucose regulation, acetic acid-treated mice exhibited lower fasting plasma glucose and HbA1c levels compared with control mice. This effect is associated with the activation of AMPK in the liver by acetate. AMPK, an energy-sensing enzyme in the liver, plays a key role in glucose homeostasis by regulating diverse metabolic processes, such as the expression of glucose transporter protein-4 (GLUT4) [151,213,214,215]. Despite its limitations by low palatability and unpleasant odor, animal as well as human studies have shown that butyrate supplementation improves insulin sensitivity, reduces diastolic blood pressure, and increases energy consumption, which may aid in managing obesity, a key risk factor for T2DM [177,210,216]. Furthermore, SCFAs have anti-inflammatory properties that help mitigate the chronic low-grade inflammation often seen in T2DM, a key driver of insulin resistance [206] (Figure 2). Additionally, SCFAs stimulate the secretion of GLP-1 and PYY, which are known for their role in insulin secretion, inhibition of glucagon secretion in the pancreas, and appetite suppression, by binding to GPRs in the gut [173,177,207]. SCFAs act by binding to the FFAR2 receptor on the surface of pancreatic β-cells, which triggers a signaling cascade that enhances insulin secretion and promotes the expansion of β-cell mass, potentially contributing to a long-term improvement in insulin sensitivity [217] (Figure 2). Recent findings also suggest that SCFAs, which are elevated in individuals with better glucose control, may represent a promising target for T2DM prevention and treatment [211]. Thus, a human study involving 60 patients with T2DM showed that treatment with sodium butyrate and inulin resulted in a decrease in fasting blood sugar in comparison with the placebo group (*p* = 0.049) [177] (Table 1). In another recent randomized clinical trial, Khosravi et al. reported decreased postprandial blood sugar levels (*p* = 0.016), as well as increased insulin levels (*p* = 0.047) and homeostatic model assessment of insulin resistance (HOMA-IR) (*p* = 0.008) in patients with T2DM treated with sodium butyrate compared to baseline [178] (Table 1).

## 4. Conclusions

In conclusion, SCFAs play an important role in the regulation of metabolic health. Through their effects on gut microbiota composition, intestinal barrier function, immune modulation, and direct metabolic signaling, SCFAs influence key pathways involved in energy homeostasis, insulin sensitivity, and inflammation. Furthermore, dysregulation of SCFA production and composition of the gut microbiome has been linked to the pathogenesis of metabolic diseases, such as MASLD, obesity, and T2DM. Therapeutic approaches involving SCFAs hold promise for the treatment of those metabolic conditions. However, despite promising preclinical and some clinical evidence, significant gaps remain in our understanding. The mechanisms underlying SCFA effects require further investigation, as does the optimal strategy for increasing SCFA levels—whether through dietary fiber, probiotics, or direct supplementation—particularly in terms of long-term safety and efficacy. Thus, additional human trials are needed to establish effective dosages, potential side effects, and interactions with existing treatments.

## Figures and Tables

**Table 1:** Clinical trials of SCFA supplementation in metabolic diseases (selected articles).

Type of Intervention	Cohort	Intervention Details	Outcome	Reference
SCFA supplementation in MASLD	50 adult patients with liver steatosis and metabolic syndrome	Randomized, double-blind, placebo-controlled clinical trial (A) (n = 25): placebo (B) (n = 25): butyrate-based formula Daily for 3 months	Fatty liver index ↓ *^,+^ Plasma triglycerides ↓ ^+^ Plasma cholesterol ↓ ^+^	Fogacci et al. [147]
SCFA supplementation in obesity (and MASLD)	60 adult patients with obesity (and MASLD)	Randomized, double-blind, placebo-controlled clinical trial (A) (n = 30): inulin (B) (n = 30): inulin-propionate Daily for 24 weeks	Postprandial plasma PYY and GLP-1 ↑ (B) * Weight gain ↓ (B) * Hepatic lipid content in patients with obesity and MASLD ↓ (B) ^+^	Chambers et al. [173]
SCFA supplementation in obesity	50 adult patients with obesity	Randomized, triple-blind, placebo-controlled clinical trial (A) (n = 25): placebo + hypo-caloric diet (B) (n = 25): sodium butyrate + hypo-caloric diet Daily for 8 weeks	Fat mass ↓ *^,+^ Visceral fat ↓ *^,+^ Fat-free mass ↑ *^,+^ Serum hs-CRP ↓ *^,+^ PBMC ADIPOR1 ↑ *^,+^ PBMC ADIPOR2 ↑ *^,+^ PBMC UCP3 ↑ *^,+^	Amiri et al. [174]
SCFA supplementation in obesity	20 adult individuals (10 healthy controls and 10 patients with obesity)	Clinical trial (A) (n = 10, healthy): sodium butyrate (B) (n = 10, obese): sodium butyrate Daily for 4 weeks	Training capacity of monocytes by oxLDL and β-glucan ↓ (B) ^+^	Cleophas et al. [175]
SCFA supplementation in pediatric obesity	54 pediatric patients with obesity	Randomized, quadruple-blind, placebo-controlled clinical trial (A) (n = 27): placebo (B) (n = 27): sodium butyrate Daily for 6 months	BMI ↓ ^+^ Waist circumference ↓ * Insulin level ↓ * HOMA-IR ↓ * Serum Ghrelin level ↓ * PBMC MicroRNA221 ↓ * Serum IL-6 level ↓ *	Coppola et al. [176]
SCFA supplementation in T2DM	60 adult patients with T2DM	Randomized, double-blind, placebo-controlled clinical trial (A) (n = 15): sodium butyrate + starch (B) (n = 15): inulin + starch (C) (n = 15): sodium butyrate + inulin (D) (n = 15): placebo Daily for 45 days	DBP ↓ (A, B, C) * Hip circumference ↓ (A) ^+^ Waist circumference ↓ (B, C) ^+^ Waist/hip ratio ↓ (C) ^+^ Fasting blood sugar ↓ (C) ^+^ GLP-1 ↑ (A, C) *	Roshanravan et al. [177]
SCFA supplementation in T2DM	42 adult patients with T2DM	Randomized, triple-blind, placebo-controlled clinical trial (A) (n = 21): placebo (B) (n = 21): sodium butyrate Daily for 6 weeks	DBP and SBP ↓ ^+^ Nitric oxide ↓ ^+^ Blood sugar 2-hr postprandial ↓ ^+^ HOMA-IR ↑ ^+^ Insulin level ↑ ^+^ Total cholesterol ↑ ^+^	Khosravi et al. [178]

* Indicates inter-group comparison between intervention and placebo group. ^+^ Indicates intra-group comparison between pre- and post-intervention. ↓ Indicates a decrease in a parameter. ↑ Indicates an increase in a parameter. ADIPOR1/2, adiponectin receptor 1/2; BMI, body mass index; DBP, diastolic blood pressure; GLP-1, glucagon-like peptide 1; HOMA-IR, homeostatic model assessment of insulin resistance; hs-CRP, high-sensitive C-reactive protein; IL-6, interleukin 6; oxLDL, oxidized low-density lipoprotein; MASLD, metabolic dysfunction-associated steatotic liver disease; PBMC, peripheral blood mononuclear cells; PYY, peptide YY; SBP, systolic blood pressure; SCFA, short-chain fatty acid; T2DM, type 2 diabetes mellitus; UCP3, uncoupling protein 3.

## Data Availability

No new data were created or analyzed in this study.

## Abbreviations

BMI, body mass index; CLD, chronic liver disease; CVD, cardiovascular disease; FFAR, free fatty acid receptors; GLP-1RA, glucagon-like peptide-1 receptor agonist; GPCR, G-protein-coupled receptor; HCC, hepatocellular carcinoma; MACE, major adverse cardiovascular events; MASH, metabolic dysfunction-associated steatohepatitis; MASLD, metabolic dysfunction-associated steatotic liver disease; NAFLD, nonalcoholic fatty liver disease; NAS, NAFLD activity score; ROS, reactive oxygen species; SCFA, short-chain fatty acid; T2DM, type 2 diabetes mellitus; THR, thyroid hormone receptor.
